# How to achieve safe, high-quality clinical studies with non-Medicinal Investigational Products? A practical guideline by using intra-bronchial carbon nanoparticles as case study

**DOI:** 10.1186/s12931-016-0413-9

**Published:** 2016-08-19

**Authors:** M. Berger, P. J. Kooyman, M. Makkee, J. S. van der Zee, P. J. Sterk, J. van Dijk, E. M. Kemper

**Affiliations:** 1 Department of Respiratory Medicine, Academic Medical Centre, University of Amsterdam, Room F5-280, Meibergdreef 9, 1105 AZ Amsterdam, The Netherlands; 2 Section Catalysis Engineering, Department of Chemical Engineering, Faculty of Applied Sciences, Delft University of Technology, Julianalaan 136, NL 2628 BL Delft, The Netherlands; 3 Department of Respiratory Diseases, Onze Lieve Vrouwe Hospital, Oosterpark 9, 1091 AC Amsterdam, The Netherlands; 4 Yellow Research, Herengracht 495, 1017 BT Amsterdam, The Netherlands; 5 Department of Pharmacy, Academic Medical Centre Amsterdam, Meibergdreef 9, 1105 AZ Amsterdam, The Netherlands

**Keywords:** Good clinical practice, Good manufacturing practice, Intervention studies, Non-medicinal products, Legislation, Guidelines

## Abstract

**Background:**

Clinical studies investigating medicinal products need to comply with laws concerning good clinical practice (GCP) and good manufacturing practice (GMP) to guarantee the quality and safety of the product, to protect the health of the participating individual and to assure proper performance of the study. However, there are no specific regulations or guidelines for non-Medicinal Investigational Products (non-MIPs) such as allergens, enriched food supplements, and air pollution components. As a consequence, investigators will avoid clinical research and prefer preclinical models or in vitro testing for e.g. toxicology studies.

**The aim of this article is to:**

1) briefly review the current guidelines and regulations for Investigational Medicinal Products; 2) present a standardised approach to ensure the quality and safety of non-MIPs in human in vivo research; and 3) discuss some lessons we have learned.

**Methods and results:**

We propose a practical line of approach to compose a clarifying product dossier (PD), comprising the description of the production process, the analysis of the raw and final product, toxicological studies, and a thorough risk-benefit-analysis. This is illustrated by an example from a human in vivo research model to study exposure to air pollutants, by challenging volunteers with a suspension of carbon nanoparticles (the component of ink cartridges for laser printers).

**Conclusion:**

With this novel risk-based approach, the members of competent authorities are provided with standardised information on the quality of the product in relation to the safety of the participants, and the scientific goal of the study.

**Electronic supplementary material:**

The online version of this article (doi:10.1186/s12931-016-0413-9) contains supplementary material, which is available to authorized users.

## Background and current legislation

Due to some tragedies concerning human medical intervention studies in the past, the research involving patients and healthy volunteers is governed by strict regulation and legislation worldwide [1–3]. The purpose is to protect the safety of the participants and ensure credible results. See Table 1 [4].

**Table 1:** Regulation and Legislation worldwide

In the U.S.A., the Food and Drug Administration (FDA) is responsible for the protection of the public health by assuring the safety, efficacy, and security of drugs, biological products, medical devices, food, and cosmetics. Based on federal legislation [4], the FDA develops several guidelines including for Good Clinical Practice (GCP).
In the European Union (EU), the European Commission (EC) is responsible for the initiation of new legislation, while the European Medicines Agency (EMA) is responsible for the scientific evaluation of medicines developed by pharmaceutical producers. The U.S.A., Japan, and the EU are also represented in the International Conference on Harmonisation of Technical Requirements for Registration of Pharmaceuticals for Human Use (ICH), which creates rules and guidelines for the development of new medicinal products.

Nowadays, the Declaration of Helsinki [5] and the Declaration of Geneva [6] represent the most important ethics policies of the World Medical Association (WMA) and are worldwide enforced. They are supported by practical guidelines focused on adequate Human Subject Protection (HSP) [5], Good Clinical Practice (GCP) [7], Good Manufacturing Practice (GMP) [8, 9], and Good Laboratory Practice (GLP) [4, 8]. See Table 2.

**Table 2:** Current practical guidelines

Good Clinical Practice (GCP)-guidelines comprise proper study design, generation of credible research data, and safety for study participants, and correct data management. These issues are assessed by a competent authority such as a medical ethics committee. Manufacturing of investigational medicinal products is highly complex. Good Manufacturing Practice (GMP) guidelines intent to ensure consistency between batches and adequate documentation of the development and production of the investigational medicinal products. The GMP Annex 13 comprises practical guidelines on the quality, production, quality control, packaging, labelling and shipping of the Active Pharmaceutical Ingredient (API) and the drug product.
In order to ensure GMP conditions, all information concerning the topics mentioned in the Annex 13 have to be documented in an Investigational Medicinal Product Dossier (IMPD). Additionally, in the EU each product batch needs to be released by a Qualified Person before the investigational medicinal product is admitted to a clinical trial.

Despite the attempt to harmonize legislation and guidelines, there are still significant differences between EU-member States’ legislation governing clinical research. One of the reasons is that some Member States feel the need to cover a broader scope than the EU Directive related to clinical research, resulting in country-specific legislation. There are also differences between the European Union and other continents, such as the U.S.A.

**Fig. 1:**
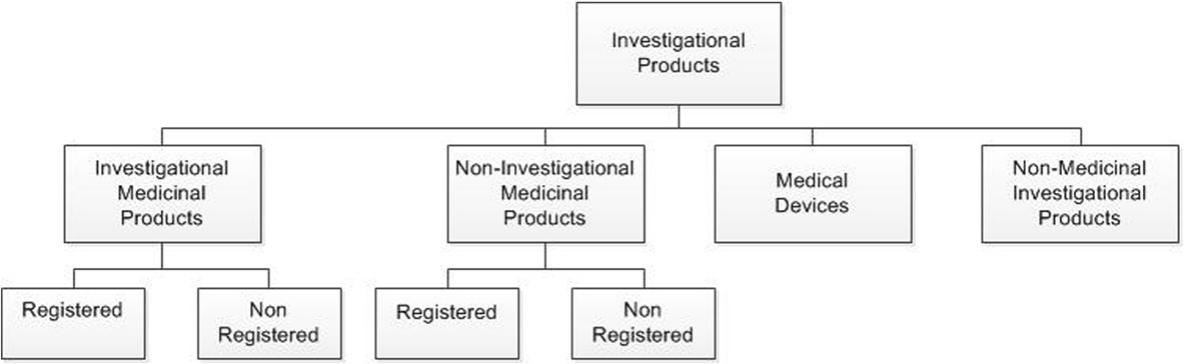
Overview of investigational products

For clinical research with both registered and non-registered medicinal products (IMPs), and non-investigational medicinal products (non-IMPs), definitions and legislation are well described [10, 11]. See Table 3 and Fig. 1.

**Table 3:** Investigational products in clinical research

- Investigational Medicinal Products (IMPs) are registered or non-registered medicines. New chemical entities, but also the medicinal product in the comparative study group and placebo’s of the challenging agents are encountered as IMPs.
- Non-Investigational Medicinal Products (non-IMPs) are medicines that are not the subject of investigation but supportive to the trial. These can include rescue medication, medicinal products given as standard care, or substances that are meant to induce a physiological response that is necessary to assess the pharmacological action of the IMP.
Medical devices: will fall outside the scope of this paper.

In contrast to the manifest guidelines for IMP and non-IMP studies, the guidelines about products that are the subject of investigation, but not regarded as a medicine, non-Medicinal Investigational Products (non-MIPs) are undefined.

These are substances used in human intervention studies to examine the physiological and toxicological effects of these compounds as opposed to investigating its pharmaceutical action. These are usually regarded as challenging agents and shared among the non-Investigational Medicinal Products (See Tables 3 and 4), with less stringent regulations as compared to Investigational Medicinal Products [8]. See Table 4.

**Table 4:** Legislation for non-investigational medicinal products

Although non-IMPs do not fall within the rules for manufacturing of investigational medicinal products, the EC does require the sponsor of a study to “ensure that the non-IMPs are in accordance with the notification/request for authorisation to conduct the trial and that they are of appropriate quality for the purposes of the trial taking into account the source of the materials, whether or not they are the subject of a marketing authorisation and whether they have been repackaged. The advice and involvement of a Qualified Person is recommended”.	

Unspecific guidelines, regulations and laws for the quality of these non-Medicinal substances represent a potential health risk to the individuals participating in trials and are causing lack of clarity to the investigators, the pharmacists, and the members of competent authorities. If the quality of the product cannot be vouched for, this can lead to unsafe research methods, even influencing the results and conclusions of a clinical trial. It is therefore difficult for the investigators to perform toxicological studies with such challenging agents.

We propose that, in order to guarantee the safety of the study subjects and the quality of the research, the investigators, who use non-Medicinal Investigational Products, will be encouraged to perform a thorough analysis and quality check of these products, including information on the raw product, the production proces of the final product, pre-clinal toxicity data, and a well-founded, product specific risk-benefit analysis. This information should be clearly documented and reviewed by the responsible competent authorities. Since these substances are not medicinal products, we are introducing an adapted Investigational Medicinal Product Dossier, simply a Product Dossier (PD), to supply Ethics Committees and Competent Authorities with adequate and sufficient information about the investigational product in a standardised way.

We will discuss the requirements for such a PD and will illustrate this with examples from the CARBON-study. Furthermore, we will share the hurdles that we have taken and lessons we have learnt to provide a helping hand when starting studies with non-Medicinal Investigational Products (non-IMPs).

## Carbon-study

### Human intervention with carbon nanoparticles

Particulate air pollution is increasingly recognised as an important causative factor in pulmonary diseases [12, 13]. To investigate the effect of carbon nanoparticles as a component of air pollution on bronchoalveolar inflammation, we aimed to develop a safe and accurate human in vivo research model. To that end, we developed a suspension of pure carbon nanoparticles, having a comparable size and structure as soot for bronchial segmental administration. The content of ink cartridges of laser printers appeared to fulfil these criteria. It is obvious that this product is not intended to be used in humans, therefore, we collected information on the raw product (characteristics and toxicity data), and thoroughly analysed the final product, in order to make a reliable risk-benefit analysis on the safety of the final product [14]. The main goals of the CARBON-study, in which we performed a bronchial segmental challenge with carbon nanoparticles in healthy volunteers, were:

1. To evaluate the safety of the study participants according to predefined criteria. Standardized endpoints were: increase in circulating leukocytes, adverse events, and complaints such as chest pain, dyspnea and cough. See also Table 5.

**Table 5:** Proposed Safety Endpoints for Bronchial Provocation Studies in Humans

Criteria	Defined by
Adverse Events	Any undesirable experience occurring to a subject during a clinical trial, whether or not considered related to the investigational product
Serious Adverse Events	Any untoward medical occurrence or effect that at any dose: - results in death; - is life threatening (at the time of the event); - requires hospitalisation or prolongation of existing inpatients’ hospitalisation; - results in persistent or significant disability or incapacity; - is a congenital anomaly or birth defect; - is a new event of the trial likely to affect the safety of the subjects, such as an unexpected outcome of an adverse reaction, major safety finding from a newly completed animal study, etc.
Blood pressure	<100/60 mmHg or > 140/90 mmHg
Heart rate	Below 50/min or above 100/min
Temperature	Below 34 °C or above 38 °C
Saturation	Below 90 %
Laboratory testing	More than a 50 % change in blood values concerning liver function, renal function, and bone marrow
FEV_1_ or PEF	Decrease of ≥ 20 %
Symptoms	e.g. Chest pain, dyspnea, cough, sore throat, dizziness and syncope

*Abbreviations: FEV1* forced expiratory volume in 1 s, *PEF* peak flow
2. To investigate the effect of carbon nanoparticles on pulmonary and systemic inflammation and coagulation. Primary endpoint was increase in local and circulating leukocytes

This study was approved by the institutional ethics committee and has been registered by the Dutch Trial Register with number 2976 at http://www.trialregister.nl/trialreg/admin/rctsearch.asp?Term=2976.

### Analysis of the raw material

A major problem with non-Medicinal substances, such as carbon nanoparticles, is that the raw material is not produced according to GMP criteria and that thorough information about the quality of the product is often not available. Furthermore, there is no information available about formation of hazardous side products, because the origin of starting materials and the way of synthesis of the raw product are not well documented or govern by trade secrets. Additional analysis of the raw product is therefore necessary and a risk-based approach is required to decide which tests are useful to gather sufficient data on the quality of the product and the suitability for its use in a clinical study. Because of the unique character of each product, we need professionals with specific knowledge of the product, and professionals with knowledge of the expected physiological action in humans to perform this thorough analysis. The next important step after finding the right product, is to perform toxicological studies in vitro and in animals. See Tables 6, 7*and the CARBON- Product Dossier* (Additional file 1).

**Table 6:** Specifications for Printex-U- suspension in saline

Test item	Method	Acceptance criteria
Description	Visual observation	Grey suspension
Primary particle size	Transmission Electron Microscopy (TEM)	< 100 nm
Ratio agglomerates <100 nm vs >100 nm	Nanoparticle Tracking Analysis (NTA)	> 50 % agglomerates/particles <100 nm
Purity	TEM/EDX	99 % carbon particles, description of the other 1 %
Arrangement	TEM	Onion-like
Contamination	TEM	No heavy metals
Microbiologic contamination	LAL-test (according to Annex 10)	< 0.1 Eu/ml

**Table 7:** CARBON-study; analysis of raw material

**Fig. 2:**
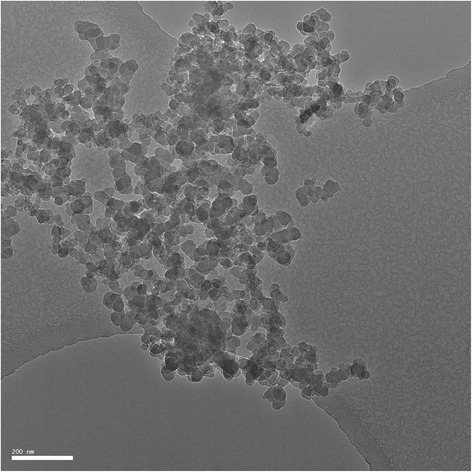
Transmission Electron Microscopic (TEM) image of Printex-U. A cluster of particles with a primary particle size of < 50 nm is shown

Carbon black pigments are the product of incomplete combustion of hydrocarbons. Depending on the specific manufacturing process a wide range of different carbon blacks are available, differing in primary particle size, structure, surface area, and surface chemistry. As these products are not intended for human use, limited information was available. We, therefore, extensively tested several commercially available products for their characteristics by transmission electron microscopy, nanoparticle tracking analysis, dynamic light scattering, and asymmetric-flow field-flow fractionation. Figure 2 shows a Transmission Electron Micrograph (TEM) of Printex-U with a cluster of particles with a primary particle size < 50 nm. Elemental analysis was performed by transmission electron microscope energy dispersive X-ray spectroscopy (TEM-EDX). The copper signal (Cu) is caused by background radiation hitting the copper sample holder grid on which the particles are deposited for measurement in the TEM. Unlike the other products, Printex-U nanoparticles had a circular shape and onion-like arrangement of atom layers comparable to that of carbon nanoparticles in diesel soot. Finally, we also tested whether our samples were contaminated with endotoxins by limulus amebocyte lysate test, which showed a contamination of < 0.01 Eu/ml [14].
Toxicological studies in literature: In vitro analysis of the raw material showed no direct mutagenic effects, but this could be secondary to other mechanisms such as oxidative stress or by triggering the inflammatory processes [22]. For these effects there was a threshold of 1 mg/m^3^ [23].
In vivo exposure to rats showed mutations in genes of the epithelial cells caused by oxidative stress. Also, in situations of impaired lung clearance (“overload”) and inflammation, some rats developed lung tumours. Mice and hamsters did not develop tumours. Various cohort and case-control studies in the U.S. did not show any increases in lung cancer among carbon black production workers [23].

### Final product: manufacturing, quality control and stability (See Table 8)

In line with regulation for medicinal products, it is important to pre-define the criteria the final product should meet. Furthermore, the manufacturing proces from raw material to final product and their associated risks should be described in detail. If possible, it is preferable to perform the proces controls.

**Table 8:** CARBON-study; analysis of final product

We claimed that the final product had to meet the following criteria: sterile, pure (no contamination with dust, LPS or metals), and the main fraction of nanoparticle aggregates had to stay nanosized when resuspended in saline (Table 6). Carbon particles tend to form firmly linked aggregates, which in turn join together to form agglomerates. In order to mimic diesel soot, it was critical for our research model that the final product contained mostly loose nanoparticles and nanoparticle aggregates smaller than 100 nm.
The final product was an isotonic suspension of carbon (Printex-U) and sodium chloride (saline) in sterile water, which was manufactured under sterile conditions (laminar airflow cabinet) in three different concentrations: 20, 100, and 200 μg carbon in 10 ml saline. The Printex-U powder was accurately weighed, mixed with pulverized sodium chloride and suspended in water for injection. After manufacturing, the product was sterilized in an autoclave (121 °C, 15 min), and sonicated for 5 min directly before administration to the study participant. Manufacturing, packaging, labelling, and batch certification was done by a qualified pharmacist.
The final product and matching placebo consisting of sterilized saline were tested for characteristics, contamination (heavy metals, dust, endotoxins), and stability.
The stability data showed an increase in particle agglomerates in time. More specifically, when analyzed 1 week after manufacturing, the main part of the particles were smaller than 100 nm, but after 1 month the number of clusters with a larger size was increased. Therefore, we decided to make a fresh sample for each study participant at a maximum of 1 week before administration on the study day.

After manufacturing, a quality control has to be performed on the final product to assess whether the product meets the pre-defined criteria. For the specifications of a product, pharmaceutical guidelines may be helpful. Next to this, as very specific analytical methods and equipment may be necessary, it is mandatory to have an agreement with the laboratory about the standard operating procedures and the quality system of the laboratory.

Stability testing is required in the development of Investigational Medicinal Products and it is usually ongoing during the development. For non-Medicinal products it is also necessary to perform stability testing in the final container to exclude formation of hazardous side products during storage and decrease in activity of the active compound.

## Product specific risk benefit analysis

In the risk-benefit-analysis the current knowledge about effects in human, animal and in vitro studies are summarised, followed by discussing (possible) mechanism of action, selectivity of the mechanism to target tissue, quality of the product, the concentration analysis, the quantitative regular daily exposure, the study design, and analysis and manageability of potential effects [15]. The investigators should also indicate how they intend to reduce the risks of the potential effects to a level that is acceptable in relation to the scientific importance of the study [16]. Dosage and route of administration are vital issues in clinical research and part of the risk-benefit analysis. A clear description is neccesary to assess safety for study participants. See Table 9 [17–19].

**Table 9:** CARBON-study; design, dosage, administration

In order to measure the single effect of the carbon nanoparticles, we selected neutral, apolar, round, onion-like, and pure carbon nanoparticles which resemble the carbon particles in air pollution concerning particle characteristics. We aimed the dosage of the final-product to be in line with normal, daily life exposure concentrations. Dosages were calculated according to the European Medicines Agency (EMA) First-In-Man (FIM) guidelines based on the No Observed Adverse Effect Level (NOAEL) in non-clinical safety studies adjusted with allometric factors. These dosages are comparable with the mean exposure concentration of PM 2.5 (particles smaller than 2.5 μm) during public fireworks at New Year’s Day at the first hour of the year, as measured by the Dutch National Air Quality Monitoring Network and previous research in the Netherlands by Strak et al. [17]
We used the well-described and safe method of bronchial segmental challenge [18, 19] to deliver the nanoparticles to the lungs. An important advantage of this model, as compared to inhalation of diesel exhaust in an inhalation chamber, is that the exposure is limited to only one component (carbon nano particles) in only one subsegment of the lung, thereby reducing the risk of generalized bronchoconstriction. In the past we safely performed bronchial segmental challenges with house dust mite allergen and lipopolysaccharide in healthy subjects as well as patients with mild asthma [18, 19]. Based upon the thorough analysis of the final product to be used and the careful considerations regarding the dosage and method of administration, the institutional ethics committee approved the study.

## Product dossier

All the collected information and test results on the product should be systematically gathered into a product dossier (PD). In line with an Investigational Medicinal Product Dossier (IMPD), this dossier should include information about the raw material, the final product, manufacturing procedures, quality control including used techniques, pharmacological data (dosage, administration route), pre-clinical toxicity data, clinical data, and a risk-benefit analysis. The PD is part of the documents submitted to the competent authorities. *See* Additional file 1*for the Product Dossier of the CARBON-study and* Table 10*for the practical checklist.*

**Table 10:** Practical checklist to prepare for clinical trials with non-medicinal investigational products

Raw material	
	Manufacturer
	Source
	Production
	Quality control (characteristics)
	Toxicity studies in vitro, animals, humans
Final product	
	Manufacturer/Pharmacist
	Pre-define the criteria the final product should meet
	Reconstitution protocols
	Sterilisation protocols
	Quality control (characteristics)
	Safety control
	Dosage/concentration analysis
	Contamination with relevant substances, e.g. endotoxion, heavy metals
	Shelf life
	Pharmacokinetics
	Toxicity studies in vitro, animals, humans
Clinical data	
	Pharmacokinetics
	Subject characteristics
	Relevant literature
Overall risk-benefit assessment	
	Administration route (reduce safety risks if possible)
	Mechanism of action (tissue specificity)
	Analysis of potential effect
	Manageability of potential effects
	Estimate the risk of side effects
	Pre-define how to manage potential effects
	Dosage (based on First-In-Man (FIM) guidelines based on the No Observed Adverse Effect Level (NOAEL) in non-clinical safety studies adjusted with allometric factors)
	Quantitative regular daily exposure
	Study design (e.g. dose-escalation or pilot study)
	Subject characteristics (medical history, age etc.)
	Appoint an independent data safety monitoring board
	Predefine safety endpoints (Table 6)
	Perform/report interim analyses on safety criteria during the study.

## Discussion and lessons learned

Research with non-Medicinal Investigational Products e.g. allergens, rhinoviruses, endotoxins, carbon nanoparticles and physiological substances such as lactate is challenging for investigators as well as members of competent authorities. An important reason is, that specific guidelines and regulations required for this type of research are lacking.

Therefore, we strongly suggest that investigators supply the Ethics Committee with a Product Dossier (PD), thereby providing standardised information about the required aspects of the non-Medicinal Investigational Product. This dossier comprises production, quality, and toxicological information about the raw material and the final product, and a risk-benefit analysis for the specific target group in the proposed study. Such a PD should be composed in collaboration with professionals equipped with product-specific and toxicological knowledge.

In light of the risk-benefit-analysis of the CARBON-study, we analysed whether carbon nanoparticles would be able to cause bronchoconstriction, pulmonary inflammation and coagulation activation to the study participants [20, 21]. We decreased these risks as much as possible by adjusting research design (escalating-dose), route of administration (localized, bronchial segmental deposition) and adjusting the dosages to relatively normal daily exposures. Naturally, safety endpoints were closely monitored and documented by the investigators and assessed by an independent Data Safety Monitoring Board (DSMB). (See Table 5).

Considering the accountability of the Ethics Committee, it is comprehensible that it has additional concerns, requests, and requirements to guarantee the safety of individuals. Fortunately, we were offered the opportunity to give further information during a face-to-face meeting with the Committee, which helped to take away major concerns.

Although we performed interim analysis after completion of each dosage-group, which was assessed by a Data Safety Monitoring Board, it would have been more conscientious if the Ethics Committee was also informed about the interim analysis. In retrospect, we also should have challenged one person per study day instead of two, in order to better monitor possible adverse reactions.

Another lesson we learned, is that the risk-benefit analysis should comprise information about pre-clinical toxicological studies on both the raw material and the final product. For the CARBON-study, there was pre-clinical toxicological information available about the raw product, but we omitted to perform these studies with the final product.

After thorough preparation, the CARBON-study was successfully completed, and showed that bronchial segmental challenge with carbon nanoparticles up to a maximum of 100 μg is safe and well tolerated.

## Conclusion

In order to guarantee safety for study participants and to ensure credible research data and harmonisation of human interventional research, we have provided a point-by-point line of approach (summarised in Table 10) for clinical trials investigating non-Medicinal substances, including instructions on how to compose a Product Dossier for the Ethics Committee to assess. One should keep in mind that each non-Medicinal Investigational Product needs its own specific, multidisciplinary analysis. With this paper we intend to draw the attention of the public and government to this issue, in order to stimulate the implementation of this line of approach as common practice for human interventional research with non-Medicinal substances.

## Abbreviations

API, active pharmaceutical ingredient; EC, European Commission; EMA, European Medicines Agency; EU, European Union; FDA, Food and Drug Administration; GCP, good clinical practice; GLP, good laboratory practice; GMP, good manufacturing practice; HSP, human subject protection; ICH, international conference on harmonisation; IMP, investigational medicinal product; IMPD, investigational medicinal product dossier; LPS, lipopolysaccharide; MA, marketing authorisation; Non-IMP, non-investigational medicinal product; Non-MIP, non-medicinal investigational product; PD, product dossier; QP, qualified person; TEM, transmission electron microscopy; TEM-EDX, transmission electron microscope-energy dispersive x-ray spectroscopy; WMA, World Medical Association; WMO, medical research involving human subjects act

## Additional file

Additional file 1: Product Dossier. (DOC 5473 kb)
